# Limited Polymorphism in the Dihydrofolate Reductase (*dhfr*) and dihydropteroate synthase genes (*dhps*) of *Plasmodium knowlesi* isolate from Thailand

**DOI:** 10.1016/j.actatropica.2023.107016

**Published:** 2023-12

**Authors:** Raweewan Sangsri, Kiattawee Choowongkomon, Runch Tuntipaiboontana, Rungniran Sugaram, Patcharida Boondej, Prayuth Sudathip, Arjen M. Dondorp, Mallika Imwong

**Affiliations:** a Department of Molecular Tropical Medicine and Genetics, Faculty of Tropical Medicine, Mahidol University, Bangkok 10400, Thailand; b Department of Biochemistry, Faculty of Science, Kasetsart University, Bangkok 10903, Thailand; c Mahidol-Oxford Tropical Medicine Research Unit, Faculty of Tropical Medicine, Mahidol University, Bangkok 10400, Thailand; d Division of Vector Borne Diseases, Department of Disease Control, Ministry of Public Health, Nonthaburi 11000, Thailand; e Centre for Tropical Medicine and Global Health, Nuffield Department of Medicine, University of Oxford, Oxford, UK

**Keywords:** Plasmodium knowlesi, pkdhfr, pkdhps

## Abstract

**Background:**

The 2022 malaria WHO reported around 4000 *P. knowlesi* infections in the South-East Asia region. In the same period, 72 positive cases were reported by the Department of Disease Control in Thailand, suggesting a persistent infection. Little is known about dihydrofolate reductase (*pkdhfr*) and dihydropteroate synthase (*pkdhps*), putative antimalarial resistance markers for *P. knowlesi*. The relevant amplification and sequencing protocol are presently unavailable. In this study, we developed a protocol for amplifying and evaluating *pkdhps* mutations. The haplotype pattern of *pkdhfr*–*pkdhps* in Thai isolates was analyzed, and the effects of these *pkdhps* mutations were predicted by using a computer program.

**Methods:**

*Pkdhps* were amplified and sequenced from 28 *P. knowlesi* samples collected in 2008 and 2020 from nine provinces across Thailand. Combining *pkdhfr* sequencing data from previous work with *pkdhps* data to analyze polymorphisms of *pkdhfr* and *pkdhps* haplotype. Protein modeling and molecular docking were constructed using two inhibitors, sulfadoxine and sulfamethoxazole, and further details were obtained through analyses of protein–ligand interactions by using the Genetic Optimisation for Ligand Docking program. A phylogenetic tree cluster analysis was reconstructed to compare the *P. knowlesi* Malaysia isolates.

**Results:**

Five nonsynonymous mutations in the *pkdhps* were detected outside the equivalence of the binding pocket sites to sulfadoxine and sulfamethoxazole, which are at N391S, E421G, I425R, A449S, and N517S. Based on the modeling and molecular docking analyses, the N391S and N517S mutations located close to the enzyme-binding pocket demonstrated a different docking score and protein–ligand interaction in loop 2 of the enzyme. These findings indicated that it was less likely to induce drug resistance. Of the four haplotypes of *pkdhfr*–*pkdhps*, the most common one is the R34L *pkdhfr* mutation and the *pkdhps* quadruple mutation (GRSS) at E421G, I425R, A449S, and N517S, which were observed in *P. knowlesi* in southern Thailand (53.57%). Based on the results of neighbor-joining analysis for *pkdhfr* and *pkdhps*, the samples isolated from eastern Thailand displayed a close relationship with Cambodia isolates, while southern Thailand isolates showed a long branch separated from the Malaysian isolates.

**Conclusions:**

A new PCR protocol amplification and evaluation of dihydropteroate synthase mutations in Knowlesi (*pkdhps*) has been developed. The most prevalent *pkdhfr*-*pkdhps* haplotypes (53.57%) in southern Thailand are R34L *pkdhfr* mutation and *pkdhps* quadruple mutation. Further investigation requires additional phenotypic data from clinical isolates, transgenic lines expressing mutant alleles, or recombinant proteins.

## Highlights

• A novel PCR protocol was developed to detect *pkdhps* polymorphisms.
• The R34L *pkdhfr* and quadruple *pkdhps* mutations are the most prevalent in Thailand.
• Molecular docking showed five *pkdhps* mutations were less likely to induce drug resistance.
• It was suggested that eastern Thailand's isolates are closely related to Cambodia.

## Graphical abstract

This is special type of abstract that is so short and could be inserted after main abstract of article, as a blurb or inserted as annotations into a Table of contents

**Figure:**
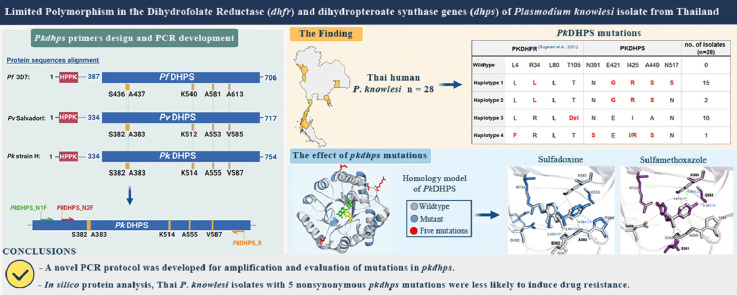

## 1 Introduction

With the implementation of a strategy for global malaria elimination by 2030, WHO reported a reduction of 80% and 77% in estimated cases and deaths in Southeast Asia in 2020 compared with 2010. Most reported malaria cases were due to *Plasmodium vivax* and *P. falciparum* (WHO, 2021). In 2004, the emergence of human infections with simian malaria (*P. knowlesi*) was reported in Malaysia Borneo (Singh et al., 2004), which was subsequently reported in other countries of Southeast Asia: Cambodia (Imwong et al., 2019), Indonesia (Figtree et al., 2010), Laos (Iwagami et al., 2018), Myanmar (Jiang et al., 2010), Singapore (Ng et al., 2008), Philippines (Luchavez et al., 2008), Vietnam (Van den Eede et al., 2010), and Thailand (Jongwutiwes et al., 2004). These incidences indicate that human *P. knowlesi* infections are more prevalent than assumed. Consequently, *P. knowlesi* is recognized as the fifth human malaria-causing agent that poses a new challenge to the global malaria elimination program (White, 2008). Long- and pig-tailed macaques (*Macaca fascicularis* and *M. nemestrina*, respectively) are natural reservoir hosts of *P. knowlesi*, which limit its distribution in Southeast Asia (Garnham, 1966). The first naturally acquired human *P. knowlesi* infection from traveling to Malaysia was reported in 1965 (Chin et al., 1965). In Thailand, *P. knowlesi* infection was reported in a patient who traveled to Prachuap Khiri Khan in 2004 (Jongwutiwes et al., 2004). *P. knowlesi* infection has also been reported in southern Thailand, where simian parasites are circulating among their natural hosts (Putaporntip et al., 2010; Putaporntip et al., 2021; Sugaram et al., 2021).

The WHO guidelines for malaria 2022 recommend artemisinin-combined therapy or chloroquine (CQ) for uncomplicated *P. knowlesi* infection and artesunate injection for complicated infection (WHO, 2022). To date, reports of *P. knowlesi* being resistant to antimalarial drugs have been lacking, and no mutations have been found in the chloroquine resistance transporter orthologous resistance genes of *P. falciparum* (Tyagi et al., 2013). Additionally, the six clinical isolates evaluated for drug susceptibility were susceptible to CQ and artemisinin and less sensitive to mefloquine (Fatih et al., 2013). Thus far, the evidence indicates that *P. knowlesi* is sensitive to antimalarial drugs.

Pyrimethamine and sulfadoxine (SDX) inhibit two critical enzymes in the folate biosynthesis pathway of parasites, namely dihydrofolate reductase (DHFR) and dihydropteroate synthase (DHPS). The molecular mechanism underlying antifolate resistance has been associated with a specific point mutation in *pf*/*pvdhfr* and *pf*/*pvdhps* genes (Korsinczky et al., 2004; Wang et al., 1997). Resistance mechanisms in *P. falciparum* and *P. vivax* have been characterized, and mutations in DHFR, and DHPS enzyme-binding pockets led to decreased drug-binding affinity (Chitnumsub et al., 2020; Yogavel et al., 2018; Yuvaniyama et al., 2003). Resistance levels have been associated with the mutation of several positions in each gene. Quadruple *pf*/*pvdhfr* mutation resulted in the highest pyrimethamine resistance level. The double mutation A437G-K540E of *pfdhps* and triple mutation S382A-A383G-A553G of *pvdhps* were found to be correlated with SDX resistance in *P. falciparum* and *P. vivax* (Japrung et al., 2007; Sirawaraporn et al., 1997; Triglia et al., 1997; Yogavel et al., 2018). Both *pf*/*pvdhfr* and *pf*/*pvdhps* mutations occur sequentially, with the first often causing a mutation in *dhfr*, followed by *dhps* when parasite populations carry at least two mutations in *dhfr (*Imwong et al., 2003*;* Lozovsky et al., 2009*;* Plowe et al., 1997*)*.

Mechanisms of antifolate resistance in *P. falciparum* and *P. vivax* are well understood, but little is known about *pkdhfr* and *pkdhps* genes in *P. knowlesi*. A few reports are available on polymorphism in *pkdhfr*, including isolates from India (Tyagi et al., 2013), Malaysia (Grigg et al., 2016), Cambodia (Imwong et al., 2019), and Thailand (Sugaram et al., 2021). By contrast, a recent study reported observing numerous *pkdhps* polymorphisms in Malaysian isolates (Saif, 2023). In addition, a protocol for *pkdhps* amplification and sequencing and information regarding the *pkdhfr*–*pkdhps* haplotype in *P. knowlesi* are lacking.

These limitations in *pkdhps*-related information represent a research gap that needs to be filled to achieve a better understanding of the biology and molecular epidemiology of drug resistance in *P. knowlesi* malaria parasites. This information can help support eliminate malaria infections in these regions due to the increasing incidence of *P. knowlesi*. In this study, seminested PCR was performed using newly designed primers to amplify the *pkdhps* gene. The frequencies and haplotype patterns of *pkdhfr*–*pkdhps* in human *P. knowlesi* infections have been reported in Thailand. The effects of observed *pkdhps* enzyme-binding site mutations were investigated through protein modeling, molecular docking, and protein interaction. Moreover, phylogenetic trees were constructed using a partial sequence of Thai *pkdhfr* and *pkdhps* genes to compare them with published Malaysian isolate data.

## 2 Material and methods

### 2.1 Study sites and sample collection

Dried blood spots of 28 symptomatic malaria were collected between 2008 and 2020 from 9 provinces of Thailand, namely Chanthaburi, Chumphon, Phang-nga, Prachuap Khiri Khan, Ranong, Surat Thani, Surin, Trat (Sugaram et al., 2021), and Tak province which included in this study (Table S1). Of these samples, 27 samples were mono-*P. knowlesi* infections, while one sample from the Tak Province was a mixed infection with *P. vivax*. All blood samples were extracted using the QIAamp® DNA Mini and Blood Mini kit (Qiagen, Germany) following the manufacturer's instructions.

### 2.2 *Pkdhps* amplification and haplotype analysis

To determine gene mutation, primers were designed, the gene was amplified, and sequences were analyzed for the partial *pkdhps* fragment. The gene locus PKNH_1429900 on NCBI was used as a reference sequence to design primers for *pkdhps* amplification by using the Primer3Plus program and OligoCalc (Kibbe, 2007; Untergasser et al., 2007). *Pkdhps* was amplified through seminested PCR and verified through Sanger sequencing (Macrogen, Korea). Although *P. knowlesi* has 80% nucleotide identity with *P. vivax*, its cross-reactivity was tested, and no cross-reactivity was observed in the primers. Table 1 presents the primers and PCR conditions used to amplify the gene. The nucleotide and amino acid sequences of these amplicons were confirmed by blasting against the NCBI database. Neutrality selection was calculated using DnaSP software within a sliding window of 100 bp and a step size of 25 bp. MEGA-X software was used to analyze point mutations by comparing the sequences generated with the reference sequences (gene locus: PKNH_1429900) and analyzing haplotypes with *pkdhfr* data from the previous study (Sugaram et al., 2021) (Table S1).

**Table 1:** Specific primers and PCR conditions were used to amplify *pkdhps* gene.

**Primers**	**Sequence (5’→3’)**	**PCR conditions**	**Product (bp)**
PkDHPS_N1F	ATGTTAACTACGATTCCTTTTCTG	nest1: 94°C for 30 s; 50°C for 30 s; 72°C for 1 min for 35 cycles.	795
PkDHPS_R	ATTTCCCTTTAGTCAGTTCTAGG		
PkDHPS_N2F	TATTCGAAATGACGAATGATGG	nest2: 94°C for 30 s; 55°C for 30 s; 72°C for 1 min for 30 cycles.	729

### 2.3 Phylogenetic trees analysis

By using a partial 649-bp sequence of *pkdhfr* from the study samples, Cambodian isolates (Imwong et al., 2019) and Malaysian isolates (Grigg et al., 2016), the neighbor-joining tree was built using the Kimura 2-parameter nucleotide substitution model and gamma distribution (K2+G), the best substitution model based on the Bayesian Information Criterion (BIC). To construct the *pkdhps* tree, partial 651-bp Thailand and Cambodia that was reported in this study and 28 extracted Malaysia datasets (Table S1) were processed using the Tamura 3-parameter base substitution method (T92+G). The *pkdhps* datasets from Malaysia were obtained from the primary genome databases. The study conducted by (Saif, 2023), utilized gene polymorphism data obtained from (Assefa et al., 2015; Pinheiro et al., 2015) as the data source to generate polymorphic samples of each Malaysian sample. Thousand bootstrap replications were used to evaluate the trees.

### 2.4 Protein homology modeling and molecular docking

The 3D structure of the *Pk*DHPS protein was modeled using the SWISS-MODEL server with default settings. The protein sequences were entered in FASTA format, and 3D homology models were retrieved as PDB files and used for molecular docking with its inhibitors.

The 3D structures of the ligands 4-aminobenzoic acid (*p*ABA), SDX, and sulfamethoxazole (SMZ) were loaded from PubChem. These ligands were used for protein docking by using the Genetic Optimisation for Ligand Docking program. Each ligand was docked within 10 Å of protein-binding site atoms and run with a genetic algorithm 100 times without the early termination. The best docking poses were selected based on the highest ASP scoring function. BIOVIA Discovery Studio Visualizer V21.1.0 analyzed the interaction of the best pose complexes. All protein structures and complexes were compared and visualized using the PYMOL Molecular Graphics System, V2.5.2.

## 3 Results

### 3.1 Polymorphism of *pkdhfr*–*pkdhps* in human isolates from Thailand

**Fig. 1:**
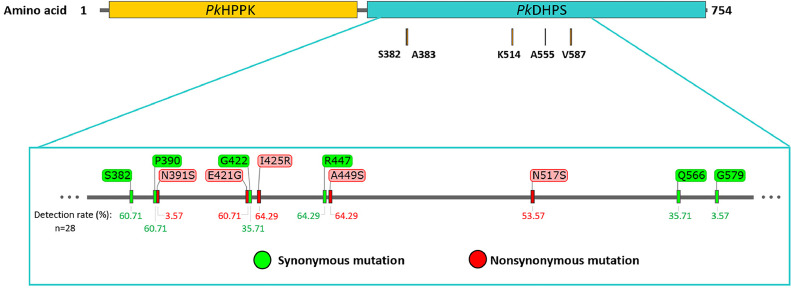
The frequency of *Pk*DHPS mutations from human *P. knowlesi* infection in Thailand and the five residues (S382, A383, K514, A555, and V587) in *Pk*DHPS that correspond to the sulfadoxine binding sites of *Pv*DHPS (S382, A383, K512, A553, V585) and *Pf*DHPS (S436, A437, K540, A581, A613) are shown.

The partial *pkdhps* of 729 bp (codon 364^th^–606^th^ of *pkhppk*-*dhps*) was successfully amplified and sequenced from 28 patients in Thailand. Eleven polymorphisms were found, which consisted of 6 synonymous and 5 nonsynonymous mutations (Fig. 1). However, these nonsynonymous mutations were not observed in the five residues of the protein–ligand binding pocket of *Pk*DHPS (namely S382, A383, K514, A555, and V587), which are equivalent to *Pv*DHPS and *Pf*DHPS that correspond to sulfa drug resistance (Table S2). A silent mutation was observed at the encoded S382 codon, which altered the third nucleotide from TCC to TCT (17/28), 60.71%, and is mainly found in the sample from southern Thailand.

Five point mutations were detected outside the enzyme-binding pocket in 18 of the 28 *pkdhps*. A quadruple mutation (GRSS) in residues E421G, I425R, A449S, and N517S was detected in isolates from southern Thailand (53.57%; 15/28): Chumphon (10/28), Ranong (1/28), Phang-nga (2/28), Surat Thani (1/8), and Prachuap Khiri Khan (1/28). The triple mutation (GRS) in the aforementioned residues, was found in 1 isolate (7.14%) each from Chumphon and Prachuap Khiri Khan. By contrast, 10 samples from the Thai-Cambodian border (35.71%; 10/28), including Surin (1/28), Trat (8/28), and Chanthaburi (1/28), were wildtypes (Table 2; Fig. S1). Furthermore, 1 sample from Tak Province had coinfection with *P. vivax* and revealed a mixed genotype of *pkdhps* at I425I/R and a unique mutation at N391S.

On combining *pkdhps* and *pkdhfr* from the previous report, four haplotype patterns appeared among the 28 clinical Thai isolates, as shown in Table 2. The two most prevalent haplotypes were haplotypes 1 (53.57%; 15/28) and 3 (35.71%; 10/28), respectively. Haplotype 1 consisted of a single R34L *dhfr*, and a quadruple *dhps* mutation was found in samples collected from southern Thailand provinces. While Haplotype 3 carried T105 deletion *dhfr*, a wildtype *pkdhps* sequence was noted in samples from provinces along the Thai-Cambodia border (Fig. S1). Four haplotypes within a nucleotide diversity (π) of 0.007 were identified across the *pkdhps* sequences of Thailand isolates, indicating the genetic diversity of Thailand isolates. A neutrality selection test statistic was observed non-significantly positive selection of Tajima's D (1.962), Fu and Li's D (0.457), and F (1.072) at *p*-value > 0.10 (Fig. S2).

**Table 2:** The *pkdhfr*-*pkdhps* haplotypes of *P. knowlesi* isolates in Thailand. Bold letters indicate the amino acid substitution and deletion mutation.

	**DHFR**^(^^Sugaram et al., 2021^^)^				**DHPS**					**Frequency (n=28)**	**Study site**
*P. falciparum*^a^ strain 3D7	V5	Y35	L81	K96	K445	**-**	**-**	A475	N543		
*P. vivax*^b^ strain SalI	L4	R34	L80	D105	S391	V421	A425	A447	N515		
*P. knowlesi*											
Wildtype^c^	L4	R34	L80	T105	N391	E421	I425	A449	N517	0% (0/28)	
Haplotype 1	L	**L**	L	T	N	**G**	**R**	**S**	**S**	53.57% (15/28)	Phang-nga, Ranong, Surat Thani, Chumphon, Prachuap Khiri Khan
Haplotype 2	L	**L**	L	T	N	**G**	**R**	**S**	N	7.14% (2/28)	Chumphon, Prachuap Khiri Khan
Haplotype 3	L	R	L	**Del**	N	E	I	A	N	35.71% (10/28)	Chanthaburi, Trat, Surin
Haplotype 4	**F**	R	L	T	**S**	E	I/**R**	**S**	N	3.57% (1/28^d^)	Tak

a Gene locus of *P. falciparum: pfdhfr* (PF3D7_0417200), *pfdhps* (PF3D7_0810800).

b Gene locus of *P. vivax: pvdhfr* (PVX_089950), *pvdhps* (PVX_123230).

c Alignment against *P. knowlesi* strain H: *pkdhfr* (PKNH_0509600), *pkdhps* (PKNH_1429900).

d *P. knowlesi* co-infected with *P. vivax*.

### 3.2 Homology *Pk*DHPS protein modeling

The effect of mutations on the inhibitor-binding sites of SDX and SMZ was determined through protein homology modeling and docking. Each point mutation N391S, E421G, I425R, A449S, and N517S was built separately into a protein model for further analysis. The built *Pk*DHPS models were based on the *Pv*DHPS crystal structure (PDB ID: 5z79) with 80.45%–80.73% sequence identity and covered 95% of the *pkdhps* sequence (Yogavel et al., 2018). Loop structure of building structures at positions 590–697 of *P. knowlesi* were disordered crystal structures of *Pv*DHPS. In addition, these *P. knowlesi* residues had two insertion sites at residues 599–626 and 675–681.

**Fig. 2:**
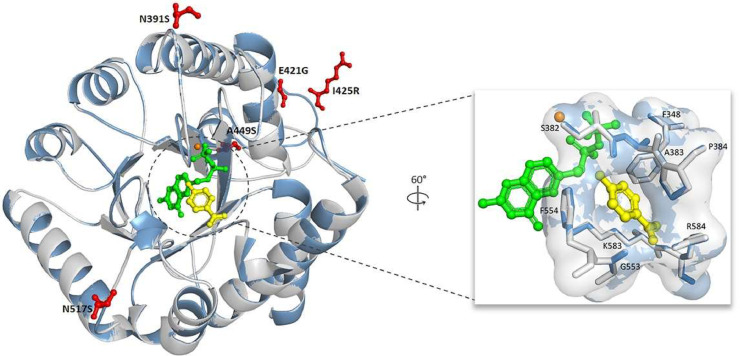
The homology model of *Pk*DHPS was constructed, and the superposition of the wildtype (light gray) and mutant *Pk*DHPS (blue) structures was annotated. It included a magnified view of the active site and the identification of contact residues, which were displayed within a compact enclosure. Furthermore, the mutations identified in this investigation are visually represented as a red-colored ball and stick model. The bound substrates *p*ABA (yellow) and PtPP (green) are depicted in a ball and stick representation, while the cofactor magnesium is represented by an orange sphere.

Among the five mutations of *Pk*DHPS, N391S, and N517S was part of the active site loop 2 and loop 5 of the enzyme, according to structural alignment against the *Pf*DHPS and *Pv*DHPS structures. N391S, located on loop 2 is involved in the flexibility conformation of the loop, causing a change in ligand–protein affinity. N517S was close to loop 5, forming a salt-bridge gate with loop 7 (Fig. 2) (Chitnumsub et al., 2020; Yogavel et al., 2018). Hence, mutants carrying these mutations were used to further characterize the effect of the inhibitor-binding affinity through protein docking.

### 3.3 Effect of mutations in *Pk*DHPS protein on inhibitor binding

**Fig. 3:**
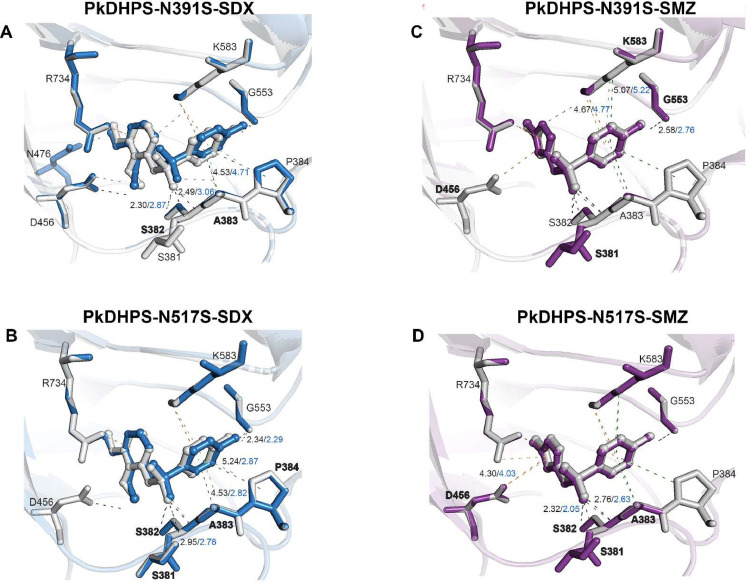
The interaction between *Pk*DHPS, sulfadoxine (SDX), and sulfamethoxazole (SMZ) has been observed. The structural components representing the interaction residues between the wildtype (gray) and mutant variants (blue; SDX; purple; SMZ) were visualized in the form of stick representations. The dashed line in the diagram represents three types of interactions: hydrogen bonds (gray), hydrophobic interactions (green), and electrostatic interactions (orange). The lighter colors within these interactions indicate interactions involving mutants. The distances between ligands and contact residues are represented by black and blue colors for the wildtype and mutant samples, respectively. The differences in interaction and distance residues between the wild-type and mutant variants are clearly indicated.

Only two residues, N391S, and N517S, were more closely located near the active site enzyme. We further characterized the effect of mutations on inhibitor binding through molecular docking within 10 Å of the active site and ran 100 times. The three ligands used in this study were the natural substrate *p*ABA and two inhibitors, SDX, and SMZ. The docking result revealed that the binding score of the N391S *Pk*DHPS mutant and ligands decreased, whereas that of the N517S mutant and ligands increased compared to the wild type (Table 3). The interaction of contact residues influences the difference in the docking score of *P. knowlesi* mutants. A loss of putative hydrogen bonds from S381 to SDX was observed in the N391S mutant. Moreover, the contact distance between residues S382, A383, and SDX increased compared to wild-type interactions, thereby reducing the docking score. By contrast, the N517S mutant revealed a reduced contact distance between SDX and residues S381, S382, A383, and P384 of *Pk*DHPS, thereby causing an increase in the docking score (Fig. 3A-B).

**Table 3:** ASP scoring function of *Pk*DHPS inhibitors and their natural substrates.

**Variants**	***p*****ABA**	**Sulfadoxine (SDX)**	**Sulfamethoxazole (SMZ)**
Wildtype *P. knowlesi*	24.23	35.35	31.88
Mutant *P. knowlesi*			
N391S	24.10 (0.13)	34.42 (0.93)	31.71 (0.17)
N517S	24.31 (-0.08)	36.03 (-0.68)	31.98 (-0.10)

Note: ASP scores have no unit and refer to the fitness of ligands. A higher score indicated a high affinity of ligands for the protein. The scores in the bracket refer to the relative docking scores.

In the *Pk*DHPS-SMZ complexes, the unique interaction of the N391S mutant with residues S381 and K583, involves hydrogen bonds, whereas the wildtype exhibits pi-cation interaction with D456. Furthermore, the distance slightly increased between the inhibitor and mutant protein residues G553 and K583 compared to the wild type. The interaction of the N517S mutant also exhibited a unique hydrogen bond interaction with residue S381. The contraction of distance between residues S382, A383, and D456 of the N517S mutant and SMZ could lead the inhibitor closer to the protein and more tight binding, resulting in an increased docking score (Fig. 3C-D). However, further experiments are required to investigate whether these mutations affect protein–inhibitor binding.

### 3.4 Phylogenetic tree analysis based on *pkdhfr* and *pkdhps* genes

**Fig. 4:**
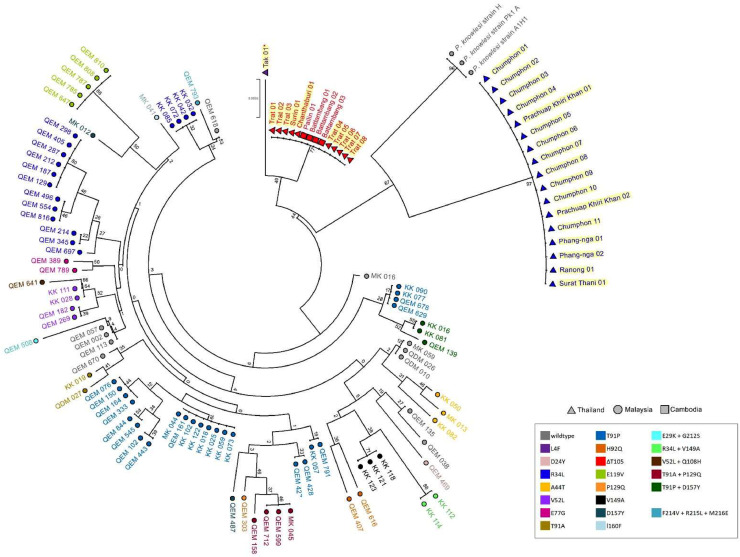
The neighbor-joining tree was constructed based on 649 bp of *pkdhfr* gene from *P. knowlesi* isolates in this study (Thailand ▲), Cambodia (■) (Imwong et al., 2019) and Malaysia (●) (Grigg et al., 2016). The colors are defined for each mutation position on the *pkdhfr* gene. The tree was supported by 1000 bootstraps. Red asterisk (*) indicated the *P. vivax* mixed infection isolate.

**Fig. 5:**
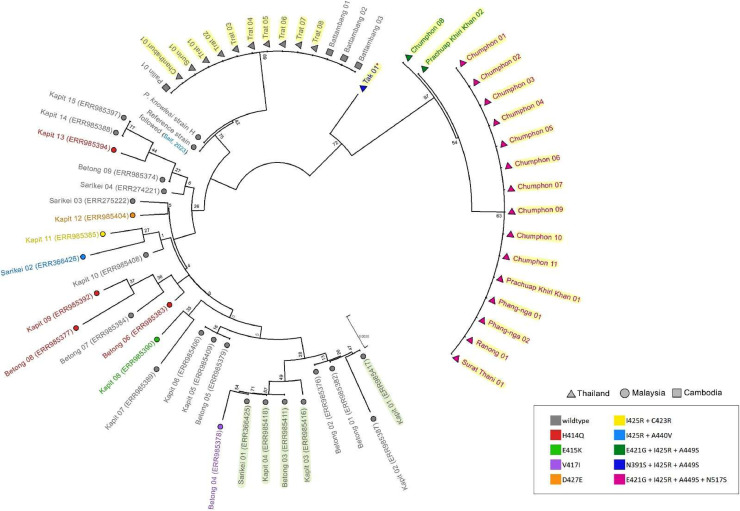
The neighbor-joining tree of the *pkdhps* gene, from samples collected in Thailand (▲), Cambodia (■), and Malaysia (●) (Saif, 2023), was constructed using a bootstrap analysis with 1000 replicates. The markers that were colored indicated the presence of a nonsynonymous mutation on the gene. A representative specimen pertaining to the *M. nemestrina* host was highlighted with a light green color. Red asterisk (*) indicated the *P. vivax* mixed infection isolates.

Phylogenetic tree cluster analysis was reconstructed to compare *P. knowlesi* from Thailand and Malaysian isolates from previous studies based on *pkdhfr* (Grigg et al., 2016) and *pkdhps* ( Saif, 2023) genes. The neighbor-joining tree is presented in Fig. 4, Fig. 5. Thailand samples isolated from Chanthaburi, Surin, and Trat were closely related to Cambodian isolates and were supported with a reliable bootstrap of 77% and 89% of *pkdhfr* and *pkdhps*, respectively, which were also observed in a previous *pkmsp1* analysis (Sugaram et al., 2021). By contrast, the southern Thailand isolates had a long branch separated from that of the Malaysian isolates, with 67% and 72% bootstrap reliability, and formed monomorphic clustering, except for 1 sample from Chumphon and Prachuap Khiri Khan provinces in the *pkdhps* tree. The sample from Tak Province was positioned differently from the clade of Thailand samples, consistent with a unique haplotype pattern. Additionally, the trees revealed sequence diversity in both genes among human Malaysian isolates.

## 4 Discussion

The National Malaria Elimination Strategy Thailand, 2017–2026, has been implemented with the vision that Thailand will be malaria-free in 2024 (Bureau of Vector Borne Diseases, 2016). Global malaria cases were reduced from 48,109 to 24,850 from 2012 to 2015 (WHO, 2021). In Thailand, malaria cases reduced from 5,432 to 3,266 between 2019 and 2021. Between 2021 and 2022, simian malaria *P. knowlesi* infection had exponentially increased from 72 to 176 cases during the COVID-19 pandemic, creating a barrier for public health staff entering the endemic areas to monitor and evaluate the disease status, including making it inconvenient for patients to enter the public health care system. Moreover, in the first quarter of 2023, the Department of Disease Control of Thailand, reported 2,103 positive malaria cases in Thailand, with *P. knowlesi* being the top two in terms of causing the highest number of infections (105/2103). The majority of the cases 92.38% (97/105) were reported from southern Thailand provinces (Division of Vector Borne, 2020). This was a 59.66% increase from positive malaria cases in 2022, indicating an increasing incidence of *P. knowlesi* infection in Thailand. Thus, this species should be acknowledged as one that needs to be controlled and eliminated from Thailand. Studies have found that *P. knowlesi* infection predominates in southern Thailand (Putaporntip et al., 2021; Sermwittayawong et al., 2012; Sugaram et al., 2021), which is close to Malaysia, where positive *P. knowlesi* infection has been predominant in humans and a high prevalence of *P. knowlesi* has also been reported in natural hosts (Akter et al., 2015; Lee et al., 2011; Saleh Huddin et al., 2019) Thus, the infection could have been transmitted to Thailand via the migration of hosts and vectors.

Attempts to study antimalarial resistance in antimalarial resistance markers of *P. knowlesi* samples have been reported in *pkcrt (*Fatih et al., 2013*;* Tyagi et al., 2013*), pkdhfr* (Grigg et al., 2016; Imwong et al., 2019; Sugaram et al., 2021; Tyagi et al., 2013), *pkkelch*, and *pkdhps* (Saif, 2023*)* genes, which are ortholog resistance genes in *P. falciparum* and *P. vivax*. A polymorphic *pkdhfr,* but not *pkdhps,* has been reported in Thailand (Sugaram et al., 2021). Moreover, a simple protocol to screen for *pkdhps* mutation is lacking. Thus, this study provided preliminary data on the status of antifolate resistance and a haplotype pattern of *pkdhfr*–*pkdhps* genes in *P. knowlesi* from Thailand by using validated PCR protocols for *pkdhps* point mutation screening. The study samples were limited because a low number of positive *P. knowlesi* infections had been detected in Thailand in the collection year 2019–2020 (Division of Vector Borne, 2020). Additional information from Thailand and other regions will be required to fill in all the gaps.

Five nonsynonymous mutations, N391S, E421G, I425R, A449S, and N517S, which were outside the *Pk*DHPS enzyme-binding site, were detected in this study. This finding indicated that *P. knowlesi* has remained susceptible to antifolate drugs and has not undergone drug selection in the parasite population in Thailand. The absence of a positive selection signal on *pkdhps*, consistent with the whole-genome sequencing results in Malaysia, indicates that its reservoir hosts have never been exposed to drug pressure, and so, no mutation exists on this resistance marker (Assefa et al., 2015). These mutations were primarily observed in southern Thailand, whereas samples from provinces along the Thai-Cambodia border contained wild-type sequences throughout the gene. A recent study revealed 73 polymorphisms throughout the *pkhppk*-*dhps* encoding bifunctional protein of *P. knowlesi* in Malaysia. Of them, 20 polymorphisms were in the residues equivalent to those observed in this study (13 synonymous and 7 nonsynonymous mutations) (Saif, 2023), except the I425R mutation, which was observed in both studies. The majority of Malaysia's genotypic pattern was wild type (19/28, 67.86%) (Table S4). Therefore, the *pkdhps* genotype of *P. knowlesi* in Thailand differed from that in Malaysia. Interestingly, the I425R mutation was detected at distinct frequencies in Thailand and Malaysia samples; however, a higher prevalence was observed in samples from southern Thailand (60.71 %; 17/28) than in samples from Malaysia (7.14 %; 2/28) (Saif, 2023). Furthermore, a single sample from Tak Province, northern Thailand, revealed a distinct *pkdhps* genotype with a mutation at residue N391S and a mixed genotype at I425I/R. Therefore, additional samples from northern Thailand should be collected to investigate this unique finding.

*The pvdhfr* quadruple mutation LRMT (F57L-S58R-T61M-S117T) increased the IC_50_ of pyrimethamine resistance by approximately 500-fold (Hastings et al., 2004), and the *pvdhps* double mutation GG (A383G-A553G) was observed to exhibit high-level SDX resistance compared to the wildtype (Yogavel et al., 2018). Combining *pvdhfr* and *pvdhps* mutations with sextuple mutations resulted in high-grade resistance, which resulted in clinical treatment failure in *P. vivax* infection (Imwong et al., 2005). In this and other studies, no mutations were observed in the predicted binding pocket. The resistance level of *P. knowlesi* was analyzed for the enzyme *Pk*DHFR but not *Pk*DHPS. At least a nanomolar concentration of pyrimethamine inhibited *Pk*DHFR, significantly influencing the schizont stage of the parasite's lifecycle (Gutteridge & Trigg, 1971). Moreover, one isolate from southern Thailand was recently characterized by an inhibitory constant (Ki) that was more sensitive to pyrimethamine than the wild type of other *Plasmodium* species (Ittarat et al., 2018), which is consistent with *in vitro* pyrimethamine testing that *P. knowlesi* revealed 10-fold drug susceptibility than *P. falciparum* (van Schalkwyk et al., 2017). Furthermore, *in vitro* testing of *Pk*DHFR inhibitors had been reported in clinical isolated from Malaysia that showed similar drug susceptibility to the lab adapted strain (van Schalkwyk et al., 2019). Additional *in vitro* and *in vivo* investigations of *Pk*DHPS requires additional research.

This study identified two *pkdhfr* mutations located outside the enzyme-binding site (Sugaram et al., 2021), namely R34L substitution (17/28; 60.71%) and T105 deletion (10/28; 35.71%), in Thai isolates. The R34L mutation was the predominant genotype detected in Thailand. It was found in samples from southern Thailand that border Malaysia, where the mutation detection rate was 8.91% (40/449), which was among the top three highest mutation detection rates in Sabah, Malaysia (Grigg et al., 2016). By contrast, the T105 deletion was identified in provinces along the Thai-Cambodia border, corresponding to data from *pkdhfr* from Cambodia's Pailin and Battambang provinces, but has never been reported from Malaysia (Table S3). This deletion, however, did not affect the *pkdhfr* frameshift (Imwong et al., 2019). Previous protein modeling studies have reported that the R34L mutation and T105 deletion were not related to drug resistance in *P. knowlesi* because the positions were far from the enzyme's binding site (Grigg et al., 2016; Sugaram et al., 2021). In addition, a novel independent mutation at L4F was identified in one sample from Tak Province, a border province between Thailand and Myanmar.

Finding the *pkdhps* mutation allowed us to identify *pkdhfr* and *pkdhps* haplotypes in Thai isolates and observe four haplotypes in our investigation. Over half of the parasites in Thailand were Haplotype 1, with the *pkdhfr* single mutation (R34L) combined with the *pkdhps* quadruple mutation (E421G, I425R, A449S, and N517S). Those samples were exclusively from southern Thailand, whereas the sample from the Thai-Cambodia border regions was Haplotype 3 (35.71%; 10/28), indicating a *pkdhfr* T105 deletion and the *pkdhps* wildtype sequence. Thus, the findings indicate that *P. knowlesi* haplotype patterns differ between geographic locations. This highest prevalence of Haplotype 1 in Thailand could be concerning, and further investigation is required to prove whether a correlation exists between these mutations and antifolate resistance in *P. knowlesi* in that part of Thailand.

In addition, the effect of quintuple mutations in *pkdhps* was evaluated through protein modeling, protein docking, and interaction analysis with *Pk*DHPS. The protein homology of *Pk*DHPS was derived on the basis of the *Pv*DHPS (PDB ID: 5z79) crystal structure and shared sequences with approximately 80% degree of similarity. Nonetheless, the *Pk*DHPS model identified loop structure at positions 590–697, which corresponded to the disordered *Pv*DHPS structure that may play a role in protein interface dimerization, but is unrelated to the binding of substrate or inhibitors, as reported in the *Pf*DHPS crystal structure (Chitnumsub et al., 2020; Yogavel et al., 2018). Differences in hydrogen bond formation and the contact distance between loop 2 (S381, S382, A383, and P384) residues of protein and the drug led to differences in the docking score of the complex of SDX with N391S and N517S mutants as well as of the complex of N517S mutant and SMZ. The distinct contact distances of the SMZ–N391S mutant at G553 and K583 were found on loops 6 and loop 7, respectively, of the binding pocket. This finding suggests that the interaction in loop 2 of the protein binding pocket had a greater impact on differences in the docking score.

The *Pv*DHPS and *Pf*DHPS crystal structures revealed that loop 2 is a flexible loop having various conformations depending on the bound ligand, which results in different ligand affinities (Chitnumsub et al., 2020; Yogavel et al., 2018). In a previous study, *Pf*DHPS residues S436, A437, and A613 were located within 3 Å of the binding site, causing a direct effect on SDX binding. Using the enzyme inhibition assays in *Pf*DHPS (S436, A437) and *Pv*DHPS (S382, A383), the critical residues of loop 2 were determined. The S436A and A437G double mutations of *Pf*DHPS resulted in a 13-fold decrease in SDX binding compared to the wild type (Chitnumsub et al., 2020), while the A383G mutation of *Pv*DHPS led to a 47-fold decrease (Pornthanakasem et al., 2016). However, the interaction between wild-type and mutant organisms revealed minor differences in this study. While computational predictions can provide valuable insights into the prediction of drug resistance mutations, they may also exhibit limitations in accurately identifying such alterations. To clarify the direct influence of these mutations on drug resistance, the inclusion of phenotypic data obtained from clinical isolates or experimental outcomes is crucial in order to validate the accuracy and dependability of these predictions.

In this study, dendrograms of Thai *pkdhfr*, and *pkdhps* demonstrated differentiated sample clusters between Peninsular and Borneo Malaysia, which is consistent with a previous finding on the single-nucleotide polymorphism (SNP) tree from whole-genome sequencing (Assefa et al., 2015). The Peninsular clade comprises most Thai isolates in the *pkdhfr* tree, but the southern Thailand isolates were assigned to a different clade in the *pkdhps* tree. Samples from the Thai-Cambodian borders shared similar genotypes with those from Cambodia, which aggregated together in the same taxa in both trees. This indicated that the migration of humans or reservoir hosts has resulted in a shared parasite population across these regions. By contrast, the southern Thailand isolates had a long branch from the Malaysian isolates, indicating that *P. knowlesi* from Malaysia could have spread to southern Thailand, which still retained ancestor characteristics and underwent little evolution. Despite this, most Thailand isolates were monomorphic, suggesting an extended migration of the parasite from other countries, which then spread in Thailand. Individual parasite population expansion in Malaysia is supported by high average nucleotide diversity in the population and the presence of individual taxa in the SNP neighbor-joining tree.

Similar to the previous report (Diez Benavente et al., 2017), the *pkdhps* tree in this study created sample clusters based on its natural hosts *M. fascicularis* (Mf-Pk) and *M. nemestrina* (Mn-Pk). However, the four Mf-Pk samples found in the Mn-Pk taxa and three of the four isolates reported genetic exchange between host-associated clusters at chromosomes 5, 8, and 11 but not at the *pkdhps* gene on chromosome 14 (Turkiewicz et al., 2023). Thus, this observation would not be a result of the parasite's genetic introgression, but rather due to the information provided by the partial gene analysis, which may be insufficient to distinguish between the two clusters. To confirm this evidence, the entire *pkdhps* gene must be analyzed and samples from Malaysia and Thailand must be compared. By contrast, host-related samples could not be indicated in the *pkdhfr* tree (Grigg et al., 2016) owing to a lack of information regarding the host association for the Malaysian isolates. To understand the fundamentals of parasite transmission from macaques to humans, the *P. knowlesi* structure in Thailand needs to be investigated to determine the relationship between the parasite's natural host and the samples.

This study fills the malaria research gap by developing and providing protocols and primers to screen *pkdhps* mutations. Moreover, the different *pkdhps* genotype frequencies between Thailand and Malaysian isolates were revealed, and this was the first study reporting the haplotype pattern of *pkdhfr*–*pkdhps* in samples from Thailand. The mutations outside the enzyme-binding pockets of *Pk*DHPS were analyzed. However, further experiments are required to investigate the effect of these mutations on antifolate drug resistance in *P. knowlesi*. However, the mutation status of other drug-resistance markers should be continually monitored. This would help malaria control programs and could limit the spread of emerging resistance parasites.

## 5 Conclusions

A novel PCR protocol was developed to amplify and evaluate mutations in dihydropteroate synthase in Knowlesi (*pkdhps*) that will be useful to fill a malaria research gap. The R34L *pkdhfr* mutation and *pkdhps* quadruple mutation are the most prevalent *pkdhfr*-*pkdhps* haplotypes (53.57%) in southern Thailand. Based on modeling and molecular docking, *P. knowlesi* isolates with five nonsynonymous *pkdhps* mutations were less likely to cause drug resistance. According to a neighbor-joining analysis, eastern Thailand isolates are closely related to Cambodia, whereas southern Thailand isolates have a long branch. Additional phenotypic data from clinical isolates, transgenic lines expressing, or recombinant proteins in the mutant alleles are required for further investigation.

## CRediT authorship contribution statement

**Raweewan Sangsri:** Methodology, Validation, Software, Data curation, Formal analysis, Visualization, Writing – original draft. **Kiattawee Choowongkomon:** Software, Formal analysis. **Runch Tuntipaiboontana:** Software, Formal analysis. **Rungniran Sugaram:** Resources. **Patcharida Boondej:** Resources. **Prayuth Sudathip:** Resources. **Arjen M. Dondorp:** Funding acquisition, Writing – review & editing. **Mallika Imwong:** Conceptualization, Investigation, Supervision, Funding acquisition, Writing – review & editing.

## Declaration of Competing Interest

The authors declare that they have no known competing financial interests or personal relationships that could have appeared to influence the work reported in this paper.

## Data Availability

Data will be made available on request.

## Appendix Supplementary materials

Image, application 1
