# Supplementary material for: Limited Polymorphism in the Dihydrofolate Reductase (dhfr) and dihydropteroate synthase genes (dhps) of Plasmodium knowlesi isolate from Thailand
Source: Acta Trop. 2023 Dec;248:107016. doi: 10.1016/j.actatropica.2023.107016 (PMC10632683; doi:10.1016/j.actatropica.2023.107016)
Supplement: Supplementary file 1 [file mmc1.docx]

**Supplementary information**

**
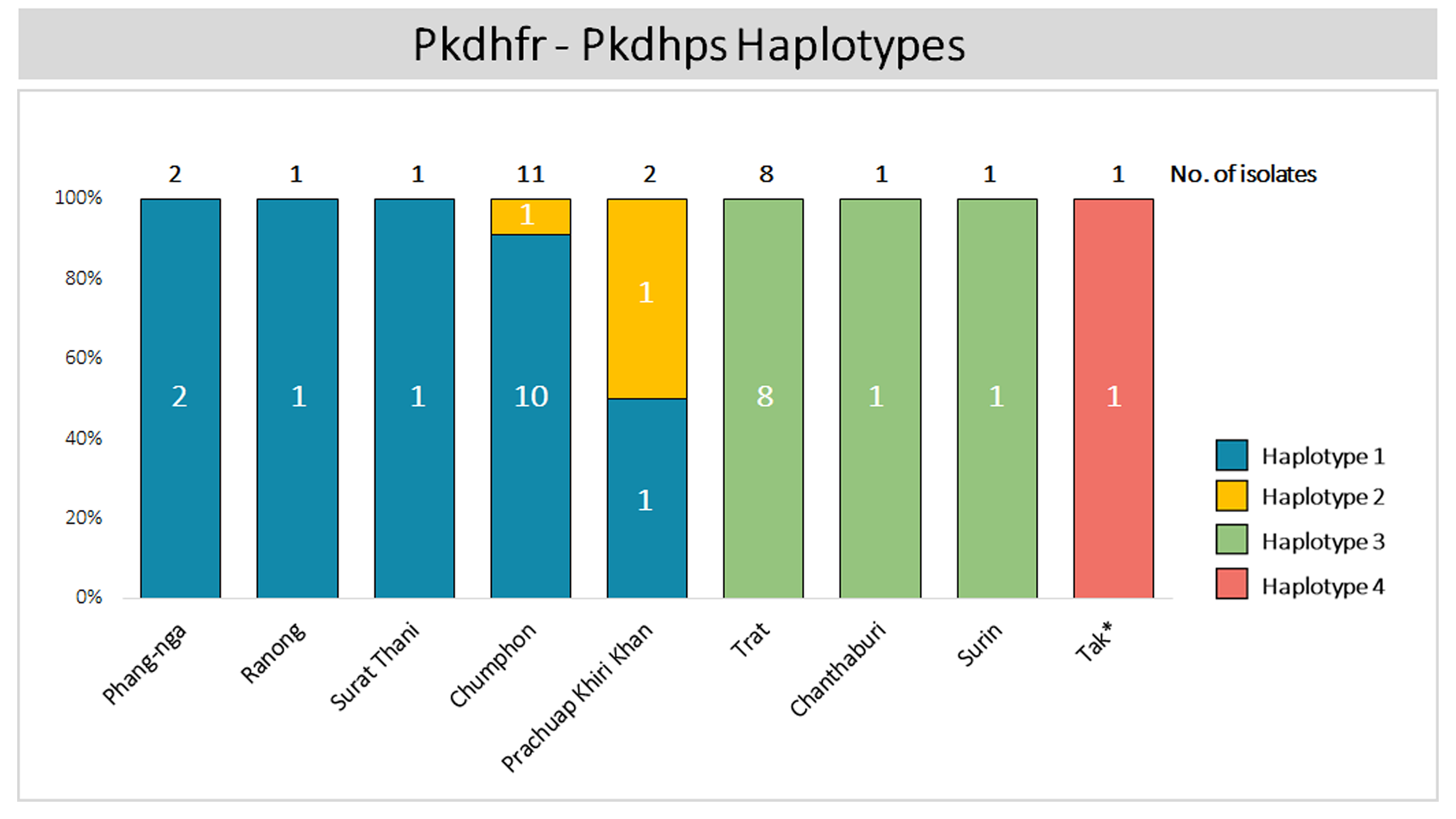
**

**Supplementary Figure S1.** Geographic distribution of *pkdhfr*-*pkdhps* haplotypes. * represented a mixed genotype at residue I425I/R and co-infection with *P*. *vivax***.**

**­**

**
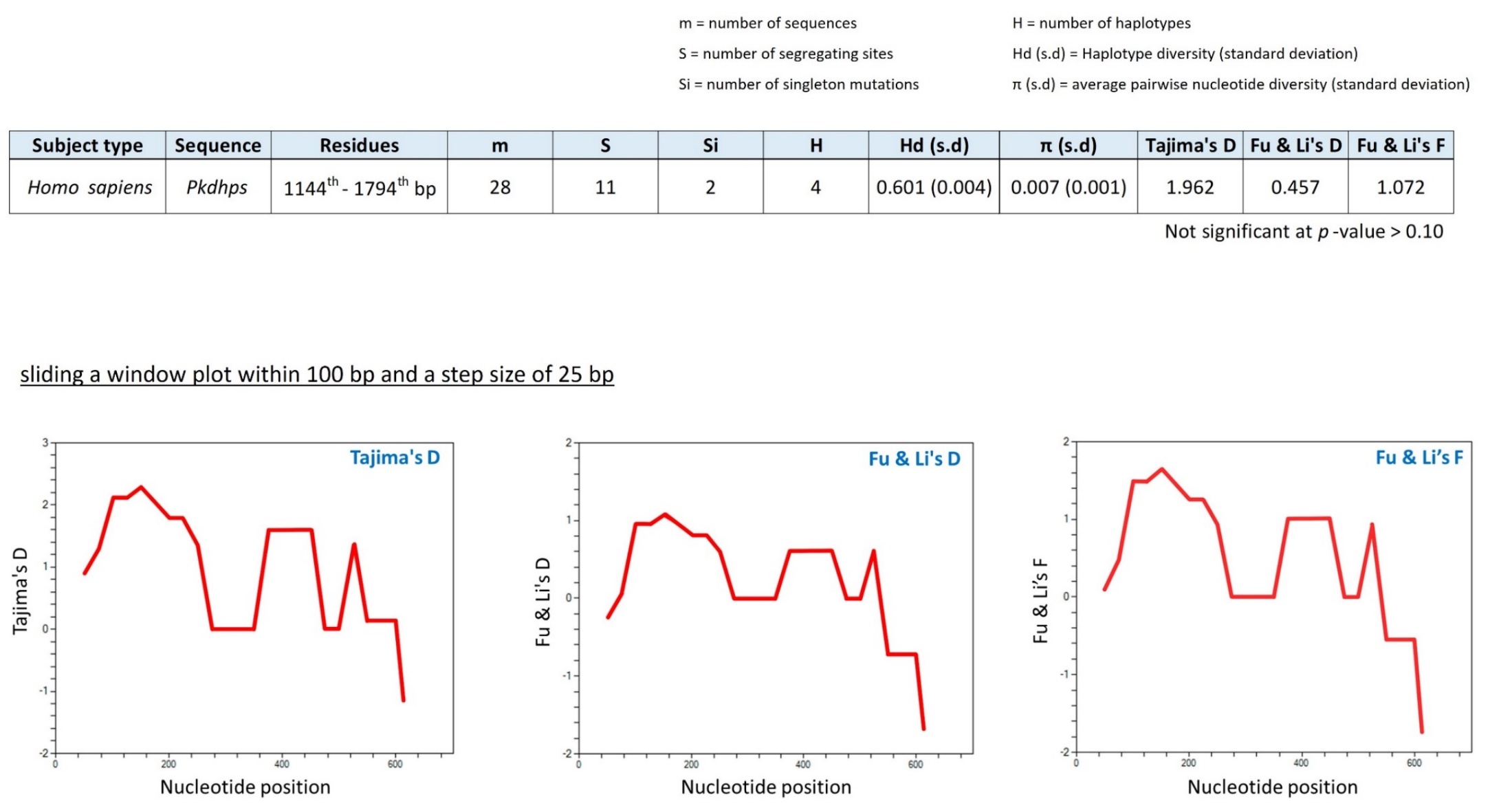
**

**­Supplementary Figure S2.** Genetic diversity and neutrality selection testing of 651 bp of *pkdhps* in human isolates from Thailand. All three statistics of neutrality selection were not significant positive value at *p*-value > 0.10.

**Supplementary Table S1**. The study site and data sources of the pkdhfr and pkdhps sequences.

| **Countries** | **Study no.** | **Study site** | **Collection year** | **N** | **Gene sequence data** | | |
| --- | --- | --- | --- | --- | --- | --- | --- |
|  |  |  |  |  | ***pkdhfr*** | | ***pkdhps*** |
| Thailand | 1 | Tak | 2008 | 1 | This study | | This study |
|  | 2 | Chumphon | 2018-2019 | 11 | (Sugaram et al., 2021) | |  |
|  | 3 | Prachuap Khiri Khan | 2019 | 2 |  |  |  |
|  | 4 | Phang-nga | 2019 | 2 |  |  |  |
|  | 5 | Ranong | 2018 | 1 |  |  |  |
|  | 6 | Surat Thani | 2019 | 1 |  |  |  |
|  | 7 | Chanthaburi | 2020 | 1 |  |  |  |
|  | 8 | Trat | 2020 | 8 |  |  |  |
|  | 9 | Surin | - | 1 |  |  |  |
| Malaysia | 1 | Sabah | 2010-2014 | 89 | (Grigg et al., 2016) | - | |
|  | 2 | Sarawak | 2012-2013 | 28 | - | (Saif, 2023) | |
| Cambodia | 1 | Pailin | 2014 | 1 | (Imwong et al., 2019) | This study | |
|  | 2 | Battambang | 2015 | 3 |  |  |  |

**Supplementary Table S2**. Equivalent binding pocket residues of PkDHFR and PkDHPS, aligned against PfDHPS and PvDHPS that well characterized to be associated with pyrimethamine and sulfa-drug resistance. The equivalent residues are marked on a schematic of the DHPR and DHPS.

|  | **DHFR residues** | | | | | | | **DHPS residues** | | | | |
| --- | --- | --- | --- | --- | --- | --- | --- | --- | --- | --- | --- | --- |
|  | 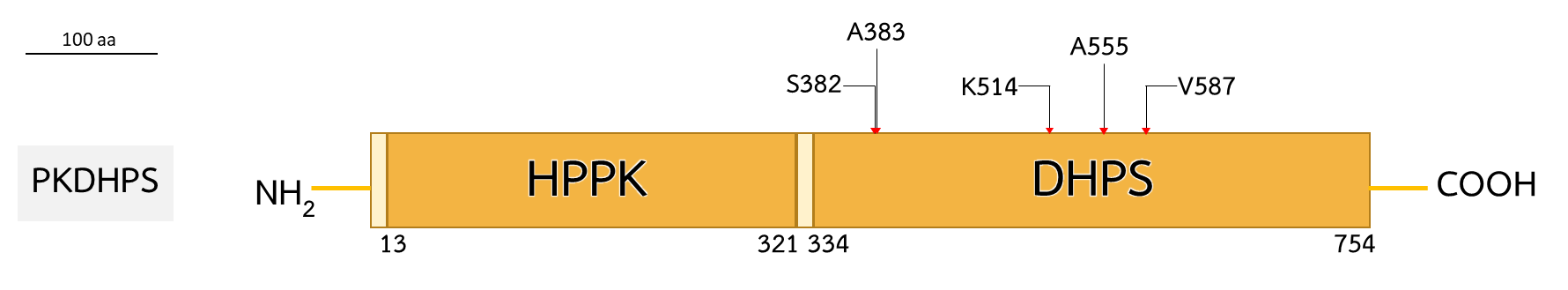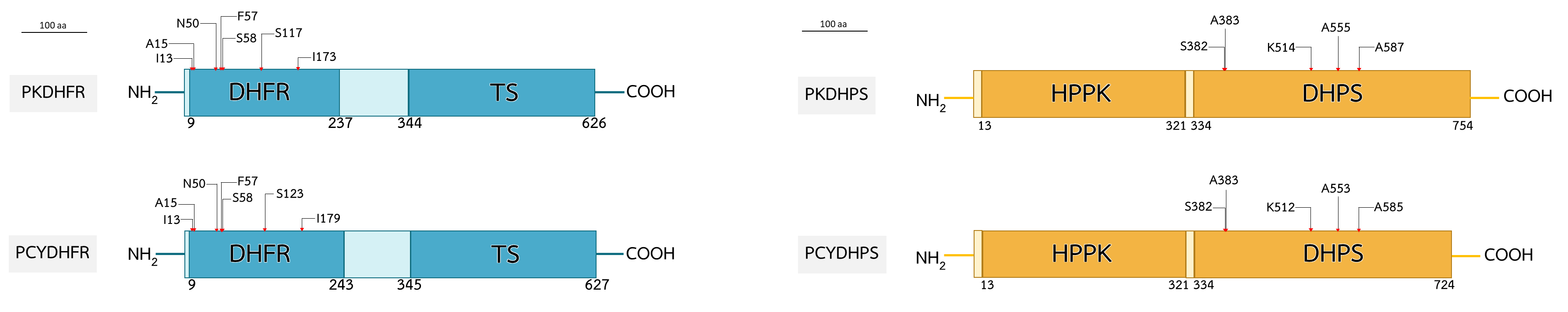 | | | | | | |  | | | | |
| **Pf**^a^ | - | A16 | N51 | - | C59 | S108 | I164 | S436 | A437 | K540 | A581 | A613 |
| **Pv**^b^ | I13 | - | - | F57 | S58 | S117 | I173 | S382 | A383 | K512 | A553 | V585 |
| **Pm**^c^ | I13 | A15 | N50 | F57 | S58 | S114 | I170 | S374 | A375 | K487 | A528 | A560 |
| **Po** | I13 | A15 | N50 | F57 | S58 | S113 | I169 | S455 | A456 | K575 | A616 | S648 |
| **Pk** | I13 | A15 | N50 | F57 | S58 | S117 | I173 | S382 | A383 | K514 | A555 | V587 |

a: data from (Plowe et al., 1997; Zolg et al., 1989)

b: data from (Imwong et al., 2001; Imwong et al., 2003; Korsinczky et al., 2004)

c: data from (Khim et al., 2012; Tanomsing et al., 2007; Tanomsing et al., 2014)

**Supplementary Table S3.** Overview of recent studies of the *pkdhfr* polymorphism.

| **no.** | **Reference** | **Countries** | **Location** | **Collecting year** | **No. of isolates *pkdhfr* amplification** | ***pkdhfr* mutation** | |
| --- | --- | --- | --- | --- | --- | --- | --- |
|  |  |  |  |  |  | **Mutation^a^** | **Prevalence (%)** |
| 1 | (Tyagi et al., 2013) | India | 1. Port Blair Island | 2003-2008 | 29 | The samples did not find any nonsynonymous mutation | |
|  |  |  | 2. Car Nicobar Island |  |  |  |  |
|  |  |  | 3. Teressa Island |  |  |  |  |
|  |  |  | 4. Nancoury island |  |  |  |  |
|  |  |  | 5. Campbell Bay Island |  |  |  |  |
| 2 | (Grigg et al., 2016) | Malaysian Borneo | 1. Kota Kinabalu district, Sabah | 2010-2014 | 449 | Wild Type | 67.71% (304/449) |
|  |  |  | 2. Kudat district, Sabah | 2012-2014 |  | T91P | 9.35% (42/449) |
|  |  |  | 3. Kota Marudu district, Sabah | 2012-2014 |  | R34L | 8.91% (40/449) |
|  |  |  |  |  |  | V149A | 3.79% (17/449) |
|  |  |  |  |  |  | A44T | 1.34% (6/449) |
|  |  |  |  |  |  | E119V | 1.34% (6/449) |
|  |  |  |  |  |  | H92Q | 1.11% (5/449) |
|  |  |  |  |  |  | V52L | 1.11% (5/449) |
|  |  |  |  |  |  | R34L, V149A | 0.89% (4/449) |
|  |  |  |  |  |  | T91A, P129Q | 0.89% (4/449) |
|  |  |  |  |  |  | T91P, D157Y | 0.67% (3/449) |
|  |  |  |  |  |  | E77G | 0.45% (2/449) |
|  |  |  |  |  |  | T91A | 0.45% (2/449) |
|  |  |  |  |  |  | T91P/A | 0.22% (1/449) |
|  |  |  |  |  |  | D157Y | 0.22% (1/449) |
|  |  |  |  |  |  | D24Y | 0.22% (1/449) |
|  |  |  |  |  |  | E29K | 0.22% (1/449) |
|  |  |  |  |  |  | L160F | 0.22% (1/449) |
|  |  |  |  |  |  | P129Q | 0.22% (1/449) |
|  |  |  |  |  |  | R34L, D157N | 0.22% (1/449) |
|  |  |  |  |  |  | T91P, P129Q | 0.22% (1/449) |
|  |  |  |  |  |  | V52L, Q108H | 0.22% (1/449) |
| 3 | (Ittarat et al., 2018) | Thailand | 1. Thai-Malaysia border | no data | 2 | Wild Type | 50% (1/2) |
|  |  |  |  |  |  | R34L | 50% (1/2) |
| 4 | (Imwong et al., 2019) | Cambodia | 1. Pailin | 2013-2014 | 1 | T105del | 100% (8/8) |
|  |  |  | 2. Battambang | 2015-2016 | 7 |  |  |
| 5 | (Sugaram et al., 2021) | Thailand | 1. Chumphon | 2018-2019 | 11 | R34L | 68.75% (11/16) |
|  |  |  | 2. Phang-nga | 2019 |  |  |  |
|  |  |  | 3. Ranong | 2018 |  |  |  |
|  |  |  | 4. Surat Thani | 2019 |  |  |  |
|  |  |  | 5. Trat | 2019-2020 | 5 | T105del | 31.25% (5/16) |
|  |  |  | 6. Surin | 2019 |  |  |  |
|  |  |  | 7. Chanthaburi | 2020 |  |  |  |

^a^aligned with reference sequence *P*. *knowlesi* strain H: (PKNH_0509600)

**Supplementary Table S4.** Overview of recent studies of the *pkdhps* polymorphism.

| **no.** | **Reference** | **No. of isolates *pkdhps* analysis** | **Countries** | **Mutation** | **Haplotype^a^** | **Prevalence (%)** | **Location** |
| --- | --- | --- | --- | --- | --- | --- | --- |
| 1 | This study | 28 | Thailand |  | Amino acid position: 391, 421, 425, 449, 517 |  |  |
|  |  |  |  | Wildtype | NEIAN | 35.71% (10/28) | Chanthaburi, Surin, Trat |
|  |  |  |  | Triple mutations***** | SERSN | 3.57% (1/28) | Tak |
|  |  |  |  | Triple mutations | NGRSN | 7.14% (2/28) | Chumphon, Prachuap Khiri Khan |
|  |  |  |  | Quadruple mutations | NGRSS | 53.57% (15/28) | Chumphon, Prachuap Khiri Khan, Phang-nga, Ranong, Surat Thani |
| 2 | (Saif, 2023) | 28 | Malaysian Borneo | Wildtype | Wildtype | 67.86% (19/28) | Kapit, Betong, Sarikei |
|  |  |  |  | Single mutation | H414Q | 14.29% (4/28) | Kapit, Betong |
|  |  |  |  |  | E415K | 3.57% (1/28) | Kapit |
|  |  |  |  |  | V417I | 3.57% (1/28) | Betong |
|  |  |  |  |  | D427E | 3.57% (1/28) | Kapit |
|  |  |  |  | Double mutations | I425R + A440V | 3.57% (1/28) | Sarikei |
|  |  |  |  |  | I425R + C423R | 3.57% (1/28) | Kapit |

Mutation residues are shown as red underlined text. ^a^aligned with the reference sequence *P*. *knowlesi* strain H: (PKNH_1429900). * indicated a sample that had co-infection with *P*. *vivax* and had mixed genotype at I425I/R.

**References**

Grigg, M. J., Barber, B. E., Marfurt, J., Imwong, M., William, T., Bird, E., et al., 2016. Dihydrofolate-Reductase Mutations in *Plasmodium knowlesi* Appear Unrelated to Selective Drug Pressure from Putative Human-To-Human Transmission in Sabah, Malaysia. PLoS One*.* 11, e0149519. <https://doi.org/10.1371/journal.pone.0149519>.

Imwong, M., Madmanee, W., Suwannasin, K., Kunasol, C., Peto, T. J., Tripura, R., et al., 2019. Asymptomatic Natural Human Infections With the Simian Malaria Parasites *Plasmodium cynomolgi* and *Plasmodium knowlesi*. J Infect Dis*.* 219, 695-702. <https://doi.org/10.1093/infdis/jiy519>.

Imwong, M., Pukrittakayamee, S., Looareesuwan, S., Pasvol, G., Poirreiz, J., White, N. J., et al., 2001. Association of genetic mutations in *Plasmodium vivax* dhfr with resistance to sulfadoxine-pyrimethamine: geographical and clinical correlates. Antimicrob Agents Chemother*.* 45, 3122-3127. <https://doi.org/10.1128/AAC.45.11.3122-3127.2001>.

Imwong, M., Pukrittayakamee, S., Renia, L., Letourneur, F., Charlieu, J. P., Leartsakulpanich, U., et al., 2003. Novel point mutations in the dihydrofolate reductase gene of *Plasmodium vivax*: evidence for sequential selection by drug pressure. Antimicrob Agents Chemother*.* 47, 1514-1521. <https://doi.org/10.1128/AAC.47.5.1514-1521.2003>.

Ittarat, W., Pornthanakasem, W., Mungthin, M., Suwandittakul, N., Leelayoova, S., Tarnchompoo, B., et al., 2018. Characterization of *Plasmodium knowlesi* dihydrofolate reductase-thymidylate synthase and sensitivity to antifolates. Parasitol Int*.* 67, 787-792. <https://doi.org/10.1016/j.parint.2018.08.004>.

Khim, N., Kim, S., Bouchier, C., Tichit, M., Ariey, F., Fandeur, T., et al., 2012. Reduced impact of pyrimethamine drug pressure on *Plasmodium malariae* dihydrofolate reductase gene. Antimicrob Agents Chemother*.* 56, 863-868. <https://doi.org/10.1128/AAC.05284-11>.

Korsinczky, M., Fischer, K., Chen, N., Baker, J., Rieckmann, K., & Cheng, Q., 2004. Sulfadoxine resistance in *Plasmodium vivax* is associated with a specific amino acid in dihydropteroate synthase at the putative sulfadoxine-binding site. Antimicrob Agents Chemother*.* 48, 2214-2222. <https://doi.org/10.1128/AAC.48.6.2214-2222.2004>.

Plowe, C. V., Cortese, J. F., Djimde, A., Nwanyanwu, O. C., Watkins, W. M., Winstanley, P. A., et al., 1997. Mutations in *Plasmodium falciparum* dihydrofolate reductase and dihydropteroate synthase and epidemiologic patterns of pyrimethamine-sulfadoxine use and resistance. J Infect Dis*.* 176, 1590-1596. <https://doi.org/10.1086/514159>.

Saif, A., 2023. Mutations in *Plasmodium knowlesi*  Kelch protein 13 and the dihydropteroate synthase gene in clinical samples. Asian Pac J Trop Biomed*.* 16, 72-79. <https://doi.org/10.4103/1995-7645.370146>.

Sugaram, R., Boondej, P., Srisutham, S., Kunasol, C., Pagornrat, W., Boonyuen, U., et al., 2021. Genetic population of *Plasmodium knowlesi* during pre-malaria elimination in Thailand. Malar J*.* 20, 454. <https://doi.org/10.1186/s12936-021-03990-x>.

Tanomsing, N., Imwong, M., Pukrittayakamee, S., Chotivanich, K., Looareesuwan, S., Mayxay, M., et al., 2007. Genetic analysis of the dihydrofolate reductase-thymidylate synthase gene from geographically diverse isolates of *Plasmodium malariae*. Antimicrob Agents Chemother*.* 51, 3523-3530. <https://doi.org/10.1128/AAC.00234-07>.

Tanomsing, N., Mayxay, M., Newton, P. N., Nosten, F., Dolecek, C., Hien, T. T., et al., 2014. Genetic variability of *Plasmodium malariae* dihydropteroate synthase (*dhps*) in four Asian countries. PLoS One*.* 9, e93942. <https://doi.org/10.1371/journal.pone.0093942>.

Tyagi, R. K., Das, M. K., Singh, S. S., & Sharma, Y. D., 2013. Discordance in drug resistance-associated mutation patterns in marker genes of *Plasmodium falciparum* and *Plasmodium knowlesi* during coinfections. J Antimicrob Chemother*.* 68, 1081-1088. <https://doi.org/10.1093/jac/dks508>.

Zolg, J. W., Plitt, J. R., Chen, G. X., & Palmer, S., 1989. Point mutations in the dihydrofolate reductase-thymidylate synthase gene as the molecular basis for pyrimethamine resistance in *Plasmodium falciparum*. Mol Biochem Parasitol*.* 36, 253-262. <https://doi.org/10.1016/0166-6851(89)90173-4>.
